# 1,10-Phenanthrolin-1-ium hydrogen d,l-tartrate dihydrate

**DOI:** 10.1107/S1600536811015972

**Published:** 2011-05-07

**Authors:** Sit Foon Cheng, Seik Weng Ng

**Affiliations:** a Department of Chemistry, University of Malaya, 50603 Kuala Lumpur, Malaysia

## Abstract

In the title hydrated molecular salt, C_12_H_9_N_2_^+^·C_4_H_5_O_6_^−^·2H_2_O, the cation is almost planar (r.m.s. deviation = 0.014 Å); the carbon skeleton of the anion assumes a *trans* conformation [C—C—C—C torsion angle = −179.86 (14)°]. The carboxyl end of one hydrogen tartrate anion forms a short hydrogen bond to the carboxyl­ate end of another anion [O⋯O = 2.508 (2) Å] in a head-to-tail manner, forming a chain; the chains and water mol­ecules inter­act, generating an O—H⋯O hydrogen-bonded layer. The cation binds to the layer by an N—H⋯O hydrogen bond.

## Related literature

For the trihydrated 1,10-phenanthrolin-1-ium salts of d- and l-tartaric acid, see: Derikvand & Olmstead (2010 ▶); Wang *et al.* (2006 ▶). 
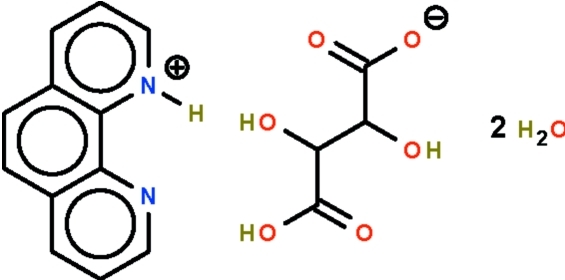


## Experimental

### 

#### Crystal data

C_12_H_9_N_2_^+^·C_4_H_5_O_6_^−^·2H_2_O
*M* *_r_* = 366.32
Triclinic, 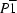

*a* = 7.0933 (7) Å
*b* = 10.5849 (11) Å
*c* = 11.4694 (11) Å
α = 98.081 (1)°
β = 100.350 (1)°
γ = 103.903 (1)°
*V* = 806.95 (14) Å^3^
*Z* = 2
Mo *K*α radiation
μ = 0.12 mm^−1^
*T* = 100 K
0.40 × 0.10 × 0.10 mm

#### Data collection

Bruker SMART APEX diffractometer
7610 measured reflections
3635 independent reflections
2880 reflections with *I* > 2σ(*I*)
*R*_int_ = 0.028

#### Refinement

*R*[*F* ^2^ > 2σ(*F* ^2^)] = 0.047
*wR*(*F* ^2^) = 0.160
*S* = 1.04
3635 reflections
267 parameters
H atoms treated by a mixture of independent and constrained refinement
Δρ_max_ = 0.35 e Å^−3^
Δρ_min_ = −0.31 e Å^−3^

### 

Data collection: *APEX2* (Bruker, 2009 ▶); cell refinement: *SAINT* (Bruker, 2009 ▶); data reduction: *SAINT*; program(s) used to solve structure: *SHELXS97* (Sheldrick, 2008 ▶); program(s) used to refine structure: *SHELXL97* (Sheldrick, 2008 ▶); molecular graphics: *X-SEED* (Barbour, 2001 ▶); software used to prepare material for publication: *publCIF* (Westrip, 2010 ▶).

## Supplementary Material

Crystal structure: contains datablocks global, I. DOI: 10.1107/S1600536811015972/bt5536sup1.cif

Structure factors: contains datablocks I. DOI: 10.1107/S1600536811015972/bt5536Isup2.hkl

Additional supplementary materials:  crystallographic information; 3D view; checkCIF report

## Figures and Tables

**Table 1:** Hydrogen-bond geometry (Å, °)

*D*—H⋯*A*	*D*—H	H⋯*A*	*D*⋯*A*	*D*—H⋯*A*
O3—H3⋯O1w^i^	0.89 (3)	1.82 (3)	2.709 (2)	175 (3)
O4—H4⋯O2w^ii^	0.90 (3)	1.84 (3)	2.739 (2)	172 (3)
O5—H5⋯O2^iii^	0.99 (3)	1.52 (3)	2.508 (2)	169 (3)
O1w—H12⋯O1	0.93 (3)	1.97 (3)	2.846 (2)	157 (3)
O1w—H11⋯O1^iv^	0.90 (4)	1.86 (4)	2.753 (2)	173 (4)
O2w—H21⋯O2	0.84 (4)	1.93 (4)	2.764 (2)	168 (3)
O2w—H22⋯O6^v^	0.87 (3)	1.97 (3)	2.835 (2)	176 (3)
N1—H1⋯O1w	0.93 (3)	1.89 (3)	2.753 (2)	153 (3)

Symmetry codes: (i) 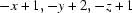; (ii) 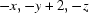; (iii) 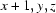; (iv) 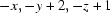; (v) 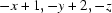.

**Fig. 1.**
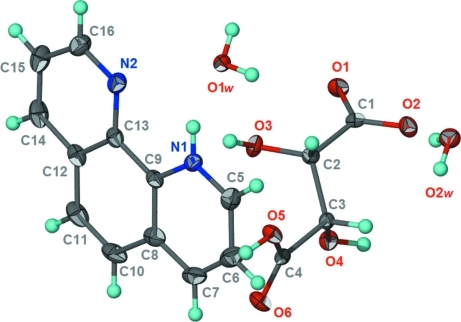
Anisotropic displacement ellipsoid plot (Barbour, 2001) of C12H9N2+ C4H5O6-.2H2O at the 70% probability level; hydrogen atoms are drawn as spheres of arbitrary radius.

**Fig. 2.**
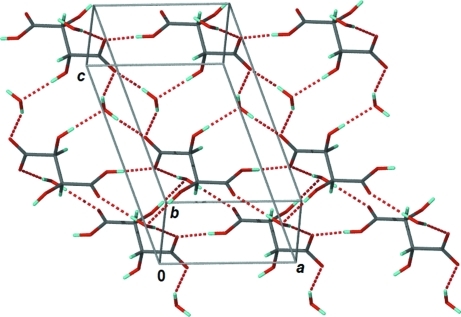
Hydrogen-bonded layer structure arising from tartrate–water interactions.

## supplementary crystallographic information

### Comment

D-tartaric acid transfers one proton to 1,10-phenanthroline to yield 1,10-phenanthroline hydrogen D-tartrate, which separates from solution as a trihydrate. The hydrogen D-tartrate anions are connected in a head-to-tail fashion by an *O*–H_carboxylic acid_···O_carboxyl_ hydrogen bond [O···O 2.455 (1) Å] (Derikvand & Olmstead, 2010). The identical feature should be presented in the L analog (Wang *et al.*, 2006). The anion and water molecules are linked by extensive O–H···O hydrogen bonds into a three-dimensional network, with the cations occupying the cavities. Racemic tartaric furnishes the corresponding dihydrate. In C_12_H_9_N_2_^+^ C_4_H_5_O_4_^-.^2H_2_O (Scheme I, Fig. 1), the carboxylic acid –CO_2_H end of one hydrogen (D,L)-tartrate anion forms a short hydrogen bond to the carboxylate –CO_2_^-^ end of another anion [O···O 2.508 (2) Å] in a head-to-tail manner to form a chain; the chains and water molecules interact to generate an O–H···O hydrogen-bonded layer. The cation binds to the layer by an N–H···O hydrogen bond (Table 1).

### Experimental

D,L-Tartaric acid (2 mmol, 0.30 g) and 1,10-phenanthroline (0.33 mmol, 0.06 g) were dissolved in water (5 ml). The solution was heated briefly to dissolve the reactants. The solution was set aside for the growth of colorless crystals, which were isolated after 10 days. The bulk crystals were faintly tinted a shade of pink.

### Refinement

Carbon-bound H-atoms were placed in calculated positions (C—H 0.95 to 1.00 Å) and were included in the refinement in the riding model approximation, with *U*(H) set to 1.2 *U*(C).

The ammonium and water H-atoms were located in a difference Fourier map, and were freely refined.

Omitted from the refinement owing to bad disagreements were these reflections: (-2 - 3 1), (-8 8 2), (2 2 4), (-7 6 7) and (3 - 3 4).

### Crystal data

**Table:**

C_12_H_9_N_2_^+^·C_4_H_5_O_6_^−^·2H_2_O	*Z* = 2
*M**_r_* = 366.32	*F*(000) = 384
Triclinic, *P*1	*D*_x_ = 1.508 Mg m^−^^3^
Hall symbol: -P 1	Mo *K*α radiation, λ = 0.71073 Å
*a* = 7.0933 (7) Å	Cell parameters from 2722 reflections
*b* = 10.5849 (11) Å	θ = 2.5–28.2°
*c* = 11.4694 (11) Å	µ = 0.12 mm^−^^1^
α = 98.081 (1)°	*T* = 100 K
β = 100.350 (1)°	Prism, colorless
γ = 103.903 (1)°	0.40 × 0.10 × 0.10 mm
*V* = 806.95 (14) Å^3^	

### Data collection

**Table:**

Bruker SMART APEX diffractometer	2880 reflections with *I* > 2σ(*I*)
Radiation source: fine-focus sealed tube	*R*_int_ = 0.028
graphite	θ_max_ = 27.5°, θ_min_ = 1.8°
ω scans	*h* = −9→9
7610 measured reflections	*k* = −13→13
3635 independent reflections	*l* = −14→14

### Refinement

**Table:**

Refinement on *F*^2^	Primary atom site location: structure-invariant direct methods
Least-squares matrix: full	Secondary atom site location: difference Fourier map
*R*[*F*^2^ > 2σ(*F*^2^)] = 0.047	Hydrogen site location: inferred from neighbouring sites
*wR*(*F*^2^) = 0.160	H atoms treated by a mixture of independent and constrained refinement
*S* = 1.04	*w* = 1/[σ^2^(*F*_o_^2^) + (0.1012*P*)^2^ + 0.1854*P*] where *P* = (*F*_o_^2^ + 2*F*_c_^2^)/3
3635 reflections	(Δ/σ)_max_ = 0.001
267 parameters	Δρ_max_ = 0.35 e Å^−^^3^
0 restraints	Δρ_min_ = −0.31 e Å^−^^3^

### Fractional atomic coordinates and isotropic or equivalent isotropic displacement parameters (Å^2^)

**Table:**

	*x*	*y*	*z*	*U*_iso_*/*U*_eq_	
O1	0.0272 (2)	0.97612 (14)	0.33939 (12)	0.0172 (3)	
O2	0.00294 (19)	1.04199 (14)	0.16223 (12)	0.0167 (3)	
O3	0.3922 (2)	0.95680 (14)	0.34847 (12)	0.0163 (3)	
O4	0.1832 (2)	0.78094 (13)	0.12086 (12)	0.0154 (3)	
O5	0.6410 (2)	1.02428 (13)	0.15147 (12)	0.0153 (3)	
O6	0.5609 (2)	0.80301 (14)	0.09605 (13)	0.0192 (3)	
O1W	0.2228 (2)	0.90933 (13)	0.55174 (12)	0.0145 (3)	
O2W	0.1595 (2)	1.23818 (15)	0.04324 (13)	0.0174 (3)	
N1	0.2289 (2)	0.65380 (16)	0.46822 (15)	0.0143 (3)	
N2	0.2713 (2)	0.66417 (16)	0.71074 (15)	0.0171 (4)	
C1	0.0952 (3)	1.00580 (17)	0.25142 (16)	0.0125 (4)	
C2	0.3085 (3)	1.00188 (18)	0.24666 (16)	0.0124 (4)	
H2A	0.3902	1.0934	0.2475	0.015*	
C3	0.3050 (3)	0.91028 (18)	0.12952 (16)	0.0115 (4)	
H3A	0.2505	0.9473	0.0594	0.014*	
C4	0.5161 (3)	0.90552 (18)	0.12418 (16)	0.0122 (4)	
C5	0.2130 (3)	0.6578 (2)	0.35202 (18)	0.0184 (4)	
H5A	0.2027	0.7366	0.3240	0.022*	
C6	0.2114 (3)	0.5459 (2)	0.27087 (19)	0.0220 (5)	
H6	0.2015	0.5487	0.1876	0.026*	
C7	0.2243 (3)	0.4319 (2)	0.31181 (19)	0.0213 (4)	
H7	0.2216	0.3552	0.2567	0.026*	
C8	0.2417 (3)	0.42852 (19)	0.43589 (19)	0.0178 (4)	
C9	0.2446 (3)	0.54419 (18)	0.51418 (17)	0.0138 (4)	
C10	0.2550 (3)	0.3129 (2)	0.4856 (2)	0.0220 (5)	
H10	0.2530	0.2340	0.4337	0.026*	
C11	0.2704 (3)	0.3145 (2)	0.6049 (2)	0.0224 (5)	
H11A	0.2778	0.2366	0.6356	0.027*	
C12	0.2757 (3)	0.43249 (19)	0.68601 (19)	0.0173 (4)	
C13	0.2645 (3)	0.54791 (18)	0.64134 (18)	0.0149 (4)	
C14	0.2935 (3)	0.4390 (2)	0.8110 (2)	0.0227 (5)	
H14	0.3004	0.3633	0.8456	0.027*	
C15	0.3006 (3)	0.5558 (2)	0.8818 (2)	0.0236 (5)	
H15	0.3135	0.5626	0.9665	0.028*	
C16	0.2888 (3)	0.6661 (2)	0.82806 (18)	0.0210 (4)	
H16	0.2936	0.7464	0.8787	0.025*	
H3	0.517 (5)	1.006 (3)	0.381 (3)	0.048 (9)*	
H4	0.066 (4)	0.767 (3)	0.067 (3)	0.032 (7)*	
H5	0.781 (5)	1.020 (3)	0.156 (3)	0.057 (10)*	
H11	0.145 (5)	0.944 (4)	0.593 (4)	0.067 (11)*	
H12	0.195 (4)	0.938 (3)	0.480 (3)	0.039 (8)*	
H21	0.120 (5)	1.172 (4)	0.074 (3)	0.052 (10)*	
H22	0.247 (5)	1.223 (3)	0.003 (3)	0.037 (8)*	
H1	0.233 (5)	0.731 (3)	0.520 (3)	0.046 (8)*	

### Atomic displacement parameters (Å^2^)

**Table:**

	*U*^11^	*U*^22^	*U*^33^	*U*^12^	*U*^13^	*U*^23^
O1	0.0162 (7)	0.0231 (7)	0.0161 (7)	0.0078 (6)	0.0087 (5)	0.0054 (5)
O2	0.0131 (7)	0.0224 (7)	0.0178 (7)	0.0070 (5)	0.0060 (5)	0.0070 (6)
O3	0.0120 (7)	0.0243 (7)	0.0118 (7)	0.0032 (6)	0.0015 (5)	0.0055 (5)
O4	0.0139 (7)	0.0137 (7)	0.0165 (7)	0.0017 (5)	0.0018 (5)	0.0020 (5)
O5	0.0123 (7)	0.0156 (7)	0.0197 (7)	0.0047 (5)	0.0058 (5)	0.0036 (5)
O6	0.0187 (7)	0.0173 (7)	0.0250 (8)	0.0097 (6)	0.0070 (6)	0.0042 (6)
O1W	0.0160 (7)	0.0181 (7)	0.0111 (7)	0.0070 (5)	0.0038 (5)	0.0037 (5)
O2W	0.0167 (7)	0.0191 (7)	0.0168 (7)	0.0047 (6)	0.0053 (6)	0.0034 (6)
N1	0.0141 (8)	0.0146 (8)	0.0132 (8)	0.0028 (6)	0.0023 (6)	0.0017 (6)
N2	0.0195 (8)	0.0153 (8)	0.0154 (8)	0.0040 (6)	0.0017 (7)	0.0033 (6)
C1	0.0139 (9)	0.0108 (8)	0.0123 (9)	0.0027 (7)	0.0044 (7)	−0.0003 (7)
C2	0.0105 (8)	0.0153 (9)	0.0120 (9)	0.0034 (7)	0.0039 (7)	0.0027 (7)
C3	0.0115 (8)	0.0142 (8)	0.0101 (8)	0.0042 (7)	0.0035 (7)	0.0041 (7)
C4	0.0146 (9)	0.0153 (8)	0.0083 (8)	0.0053 (7)	0.0036 (7)	0.0041 (7)
C5	0.0159 (9)	0.0225 (10)	0.0166 (10)	0.0039 (8)	0.0040 (7)	0.0051 (8)
C6	0.0196 (10)	0.0278 (11)	0.0169 (10)	0.0038 (8)	0.0059 (8)	0.0009 (8)
C7	0.0148 (9)	0.0226 (10)	0.0229 (11)	0.0041 (8)	0.0051 (8)	−0.0062 (8)
C8	0.0110 (9)	0.0167 (9)	0.0230 (10)	0.0016 (7)	0.0039 (7)	−0.0015 (8)
C9	0.0107 (8)	0.0125 (8)	0.0178 (10)	0.0037 (7)	0.0020 (7)	0.0015 (7)
C10	0.0184 (10)	0.0128 (9)	0.0330 (12)	0.0042 (8)	0.0063 (9)	−0.0019 (8)
C11	0.0201 (10)	0.0122 (9)	0.0354 (12)	0.0058 (8)	0.0042 (9)	0.0065 (8)
C12	0.0131 (9)	0.0145 (9)	0.0239 (11)	0.0038 (7)	0.0020 (8)	0.0050 (8)
C13	0.0113 (8)	0.0134 (9)	0.0189 (10)	0.0029 (7)	0.0016 (7)	0.0031 (7)
C14	0.0227 (10)	0.0194 (10)	0.0282 (11)	0.0068 (8)	0.0026 (9)	0.0137 (9)
C15	0.0270 (11)	0.0248 (11)	0.0188 (10)	0.0056 (9)	0.0016 (8)	0.0105 (8)
C16	0.0262 (11)	0.0180 (10)	0.0164 (10)	0.0047 (8)	0.0016 (8)	0.0017 (8)

### Geometric parameters (Å, °)

**Table:**

O1—C1	1.241 (2)	C3—H3A	1.0000
O2—C1	1.269 (2)	C5—C6	1.395 (3)
O3—C2	1.410 (2)	C5—H5A	0.9500
O3—H3	0.89 (3)	C6—C7	1.372 (3)
O4—C3	1.412 (2)	C6—H6	0.9500
O4—H4	0.91 (3)	C7—C8	1.412 (3)
O5—C4	1.311 (2)	C7—H7	0.9500
O5—H5	1.00 (3)	C8—C9	1.406 (3)
O6—C4	1.219 (2)	C8—C10	1.437 (3)
O1W—H11	0.90 (4)	C9—C13	1.434 (3)
O1W—H12	0.92 (3)	C10—C11	1.350 (3)
O2W—H21	0.84 (4)	C10—H10	0.9500
O2W—H22	0.87 (3)	C11—C12	1.437 (3)
N1—C5	1.325 (3)	C11—H11A	0.9500
N1—C9	1.360 (3)	C12—C13	1.403 (3)
N1—H1	0.93 (3)	C12—C14	1.407 (3)
N2—C16	1.326 (3)	C14—C15	1.365 (3)
N2—C13	1.354 (2)	C14—H14	0.9500
C1—C2	1.534 (2)	C15—C16	1.407 (3)
C2—C3	1.535 (2)	C15—H15	0.9500
C2—H2A	1.0000	C16—H16	0.9500
C3—C4	1.522 (2)		
			
C2—O3—H3	111 (2)	C5—C6—H6	120.1
C3—O4—H4	110.2 (17)	C6—C7—C8	119.99 (19)
C4—O5—H5	111.6 (19)	C6—C7—H7	120.0
H11—O1W—H12	101 (3)	C8—C7—H7	120.0
H21—O2W—H22	109 (3)	C9—C8—C7	118.09 (19)
C5—N1—C9	122.93 (18)	C9—C8—C10	118.62 (19)
C5—N1—H1	117.6 (19)	C7—C8—C10	123.30 (19)
C9—N1—H1	119.4 (19)	N1—C9—C8	119.30 (18)
C16—N2—C13	116.48 (17)	N1—C9—C13	119.82 (17)
O1—C1—O2	125.36 (18)	C8—C9—C13	120.88 (18)
O1—C1—C2	119.50 (17)	C11—C10—C8	121.05 (19)
O2—C1—C2	115.13 (16)	C11—C10—H10	119.5
O3—C2—C1	109.60 (14)	C8—C10—H10	119.5
O3—C2—C3	110.78 (15)	C10—C11—C12	120.86 (19)
C1—C2—C3	109.30 (14)	C10—C11—H11A	119.6
O3—C2—H2A	109.0	C12—C11—H11A	119.6
C1—C2—H2A	109.0	C13—C12—C14	117.29 (18)
C3—C2—H2A	109.0	C13—C12—C11	119.93 (19)
O4—C3—C4	109.91 (14)	C14—C12—C11	122.78 (18)
O4—C3—C2	111.42 (14)	N2—C13—C12	124.24 (18)
C4—C3—C2	109.66 (14)	N2—C13—C9	117.11 (17)
O4—C3—H3A	108.6	C12—C13—C9	118.65 (18)
C4—C3—H3A	108.6	C15—C14—C12	118.92 (19)
C2—C3—H3A	108.6	C15—C14—H14	120.5
O6—C4—O5	124.72 (17)	C12—C14—H14	120.5
O6—C4—C3	123.46 (17)	C14—C15—C16	119.4 (2)
O5—C4—C3	111.81 (15)	C14—C15—H15	120.3
N1—C5—C6	119.94 (19)	C16—C15—H15	120.3
N1—C5—H5A	120.0	N2—C16—C15	123.7 (2)
C6—C5—H5A	120.0	N2—C16—H16	118.1
C7—C6—C5	119.74 (19)	C15—C16—H16	118.1
C7—C6—H6	120.1		
			
O1—C1—C2—O3	2.5 (2)	C10—C8—C9—C13	1.3 (3)
O2—C1—C2—O3	−178.33 (15)	C9—C8—C10—C11	−0.1 (3)
O1—C1—C2—C3	124.08 (18)	C7—C8—C10—C11	−179.65 (19)
O2—C1—C2—C3	−56.7 (2)	C8—C10—C11—C12	−0.6 (3)
O3—C2—C3—O4	62.92 (19)	C10—C11—C12—C13	0.1 (3)
C1—C2—C3—O4	−57.94 (19)	C10—C11—C12—C14	−179.4 (2)
O3—C2—C3—C4	−58.99 (19)	C16—N2—C13—C12	−0.3 (3)
C1—C2—C3—C4	−179.86 (14)	C16—N2—C13—C9	179.70 (17)
O4—C3—C4—O6	11.4 (2)	C14—C12—C13—N2	0.6 (3)
C2—C3—C4—O6	134.17 (19)	C11—C12—C13—N2	−178.96 (18)
O4—C3—C4—O5	−169.64 (14)	C14—C12—C13—C9	−179.49 (17)
C2—C3—C4—O5	−46.8 (2)	C11—C12—C13—C9	1.0 (3)
C9—N1—C5—C6	0.1 (3)	N1—C9—C13—N2	−1.5 (3)
N1—C5—C6—C7	0.7 (3)	C8—C9—C13—N2	178.28 (17)
C5—C6—C7—C8	−0.8 (3)	N1—C9—C13—C12	178.54 (17)
C6—C7—C8—C9	0.2 (3)	C8—C9—C13—C12	−1.7 (3)
C6—C7—C8—C10	179.72 (19)	C13—C12—C14—C15	−0.6 (3)
C5—N1—C9—C8	−0.8 (3)	C11—C12—C14—C15	178.92 (19)
C5—N1—C9—C13	179.01 (18)	C12—C14—C15—C16	0.4 (3)
C7—C8—C9—N1	0.6 (3)	C13—N2—C16—C15	0.1 (3)
C10—C8—C9—N1	−178.97 (17)	C14—C15—C16—N2	−0.2 (3)
C7—C8—C9—C13	−179.19 (17)		

### Hydrogen-bond geometry (Å, °)

**Table:**

*D*—H···*A*	*D*—H	H···*A*	*D*···*A*	*D*—H···*A*
O3—H3···O1w^i^	0.89 (3)	1.82 (3)	2.709 (2)	175 (3)
O4—H4···O2w^ii^	0.90 (3)	1.84 (3)	2.739 (2)	172 (3)
O5—H5···O2^iii^	0.99 (3)	1.52 (3)	2.508 (2)	169 (3)
O1w—H12···O1	0.93 (3)	1.97 (3)	2.846 (2)	157 (3)
O1w—H11···O1^iv^	0.90 (4)	1.86 (4)	2.753 (2)	173 (4)
O2w—H21···O2	0.84 (4)	1.93 (4)	2.764 (2)	168 (3)
O2w—H22···O6^v^	0.87 (3)	1.97 (3)	2.835 (2)	176 (3)
N1—H1···O1w	0.93 (3)	1.89 (3)	2.753 (2)	153 (3)

Symmetry codes: (i) −*x*+1, −*y*+2, −*z*+1; (ii) −*x*, −*y*+2, −*z*; (iii) *x*+1, *y*, *z*; (iv) −*x*, −*y*+2, −*z*+1; (v) −*x*+1, −*y*+2, −*z*.
